# PerSeqPIPE: Nextflow workflow for per-sequence analysis of small RNA-sequencing data

**DOI:** 10.1093/bioadv/vbag294

**Published:** 2026-10-05

**Authors:** Karolína Trachtová, Dagmar Al Tukmachi, Vojtěch Bystrý, Ondřej Slabý

**Affiliations:** Central European Institute of Technology, Masaryk University, 62500 Brno, Czech Republic; National Centre for Biomolecular Research, Faculty of Science, Masaryk University, 62500 Brno, Czech Republic; Central European Institute of Technology, Masaryk University, 62500 Brno, Czech Republic; Central European Institute of Technology, Masaryk University, 62500 Brno, Czech Republic; Central European Institute of Technology, Masaryk University, 62500 Brno, Czech Republic; Department of Biology, Faculty of Medicine, Masaryk University, 62500 Brno, Czech Republic; Center for Precision Molecular Diagnostics, Masaryk Memorial Cancer Insitute, 65653 Brno, Czech Republic

## Abstract

**Summary:**

Small RNA sequencing enables high-throughput profiling of diverse small non-coding RNAs, but their complex biology creates substantial bioinformatic challenges. Conventional gene-centric pipelines often discard multi-mapping reads or impose arbitrary feature assignments, leading to biased quantification results. We present PerSeqPIPE, a modular, fully containerized Nextflow pipeline implementing a sequence-centric approach that quantifies each unique read sequence and annotates it with all compatible genomic and transcriptomic features. This preserves biologically relevant signals and enables broad small non-coding RNA profiling. PerSeqPIPE integrates harmonized reference resources for multiple small non-coding RNA classes and supports reproducible execution using Docker and Apptainer. The accompanying Streamlit-based PerSeqPIPE VIZ application enables interactive exploration, filtering, visualization, and differential expression analysis. Application to a published extracellular vesicle dataset revealed isomiR diversity and multi-mapped sncRNAs that were obscured by conventional approaches. Together, PerSeqPIPE and PerSeqPIPE VIZ provide an open, scalable, and transparent framework for small RNA-seq analysis.

**Availability and implementation:**

PerSeqPIPE is freely available at https://github.com/ktrachtova/perseqpipe and PerSeqPIPE VIZ at https://github.com/ktrachtova/perseqpipe_viz. PerSeqPIPE is implemented in Nextflow, Python, Bash, and R; PerSeqPIPE VIZ is a Streamlit app. Both are compatible with macOS and Linux operating systems.

## 1 Introduction

Small RNA sequencing (small RNA-seq) enables high-throughput profiling of small non-coding RNAs (sncRNAs), including microRNAs (miRNAs), isomiRs, piwi-interacting RNAs (piRNAs), small nucleolar RNAs (snoRNAs), and tRNA-derived fragments. These sncRNAs play crucial roles in physiological and pathological processes and are often identified as biomarkers (Zhang *et al.* 2021).

Unfortunately, bioinformatic analysis of small RNA-seq data remains challenging because sncRNAs are short, frequently exist as multiple isoforms, originate from multiple genomic loci, or overlap other transcripts (Borchert *et al.* 2011, Wright *et al.* 2022). Small RNA-seq libraries may also contain fragments of mRNAs, lncRNAs, and rRNAs, particularly in body fluids or degraded samples (Godoy *et al.* 2018).

Several tools address specific aspects of small RNA analysis. miRNAs and their sequence variants, commonly referred to as isomiRs, are comparatively well studied, with established naming conventions and tools for their detection and quantification (Pantano *et al.* 2010). In contrast, analysis of other sncRNA classes remains less standardized. Tools like OASIS or COMPSRA support broader multi-class annotation, but they mostly rely on gene-centric quantification strategies where multi-mapping reads are discarded, fractionally assigned, or resolved using predefined class priority rules (Capece *et al.* 2015, Li *et al.* 2020). Although this simplifies interpretation, it can underestimate multi-locus sncRNAs and obscure the complexity of reads that legitimately map to multiple genomic features. Furthermore, many tools present challenges for reproducible deployment and integration into larger analysis workflows.

Here, we present PerSeqPIPE, a modular and fully containerized Nextflow pipeline for comprehensive small RNA-seq analysis. PerSeqPIPE combines broad sncRNA profiling with sequence-centric quantification, in which each distinct read sequence is quantified once and annotated with all compatible genomic and transcriptomic features. The pipeline is distributed with containerized dependencies and a precompiled genome-coordinated annotation integrating multiple sncRNA databases, while also supporting user-provided custom annotations. PerSeqPIPE outputs can be interactively explored using the accompanying Streamlit-based PerSeqPIPE VIZ application.

## 2 Results

### 2.1 Pipeline overview

PerSeqPIPE is a modular Nextflow pipeline that provides a one-stop framework for small RNA-seq data analysis (Fig. 1), with inputs and parameters defined in the configuration file or directly from the command line. Default parameters for the tools used are provided in Table S1, available as supplementary data at *Bioinformatics Advances* online.

**Figure 1:**
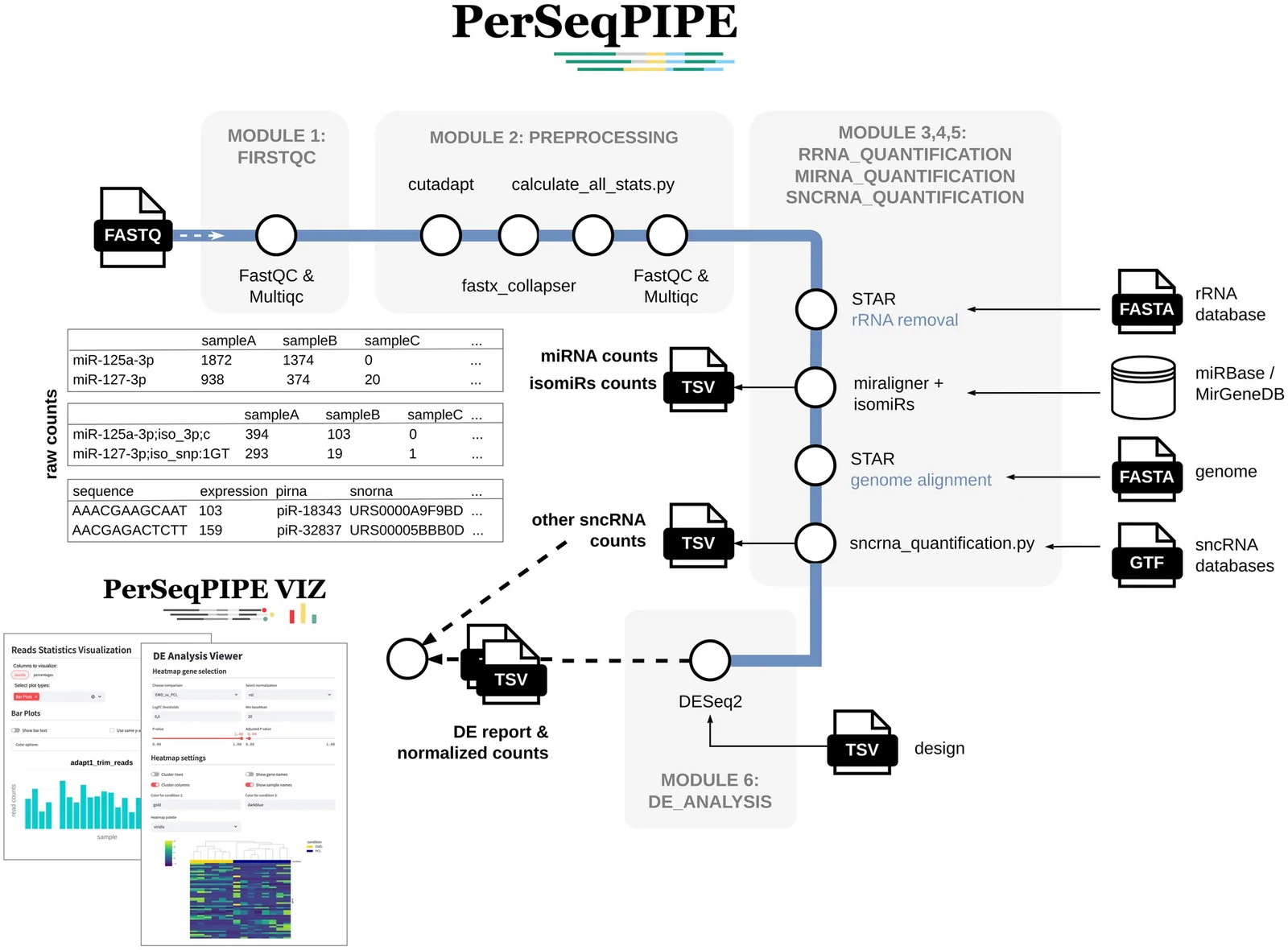
Data flow through the PerSeqPIPE workflow. FASTQ files provided via a samplesheet undergo preprocessing, during which sequences are cleaned, collapsed, and assigned to sncRNA classes using three quantification modules. Each quantification module requires a class-specific reference: an rRNA FASTA file for removal of rRNA contamination, the miRBase or MirGeneDB database for miRNA/isomiR quantification, and a GTF file for other sncRNAs. The final module requires a design file to perform statistical comparisons between conditions. The PerSeqPIPE workflow can be executed as a whole, or only specific modules can be selected using flow-control parameters. Output files can be loaded into the PerSeqPIPE VIZ application for interactive visualization of read processing statistics, quantified sncRNAs, and statistical analysis results.

PerSeqPIPE consists of six main modules, each implemented as a Nextflow subworkflow, covering initial quality assessment, preprocessing, rRNA contamination removal, quantification of miRNAs and isomiRs, quantification of other sncRNAs and mRNA/lncRNA fragments, and differential expression analysis.

The first two modules, FirstQC and Preprocessing, perform an initial quality assessment and cleaning of raw FASTQ files. Preprocessing steps are automatically selected based on the user-specified library preparation kit (supported kits are listed in Table S2, available as supplementary data at *Bioinformatics Advances* online).

Three quantification modules assess rRNA contamination, miRNA/isomiR expression, and the expression of other sncRNAs. Canonical miRNA and isomiR quantification is performed separately from other sncRNAs as their biology requires a specific approach, considering their 5’ and 3’ end nucleotide modifications and established naming system (Desvignes *et al.* 2020).

The final module performs differential expression analysis using a user-provided design file, reporting statistics such as log2 fold changes, adjusted *P*-values, and expression levels across samples.

Read counts are tracked throughout preprocessing and quantification, supporting quality assessment and full data traceability; each reported sequence can be traced back to the raw FASTQ file.

PerSeqPIPE leverages Nextflow’s native parallelization and caching to support scalable execution across local, cluster, and cloud environments. The default configuration requires up to 4 CPUs and 16 GB RAM for individual processes, while systems with 32 CPUs and 64 GB RAM are recommended to enable efficient parallel processing of multiple samples. Further benchmarking of runtime, CPU and memory usage, storage requirements, and performance scaling with increasing sample batch size is provided in Table S3, available as supplementary data at *Bioinformatics Advances* online.

### 2.2 Details on sequence-centric sncRNA quantification

The core component of PerSeqPIPE is a sequence-centric quantification approach. After alignment of reads to the genome, an in-house Python script assigns all compatible annotations to each read. In contrast to traditional gene-centric approaches that may discard or fractionally redistribute ambiguously mapped reads (Anders *et al.* 2015), PerSeqPIPE counts each distinct aligned sequence once while retaining all compatible annotations. Sequence-centric quantification with PerSeqPIPE can also identify novel RNAs, as reads lacking known annotations are also retained.

However, this approach also involves trade-offs that users should consider. Per-sequence resolution generates substantially more features than gene-centric methods, increasing the multiple testing burden in differential expression analysis. Technical artifacts—sequencing errors, library preparation artifacts, and degradation products—are quantified as distinct sequences alongside genuine biological variants. Therefore, parameters such as minimum sequence expression and required read–feature overlap are fully configurable, allowing users to filter low-abundance sequences and adjust annotation stringency.

The sncRNA quantification output reports per-sequence expression, all compatible annotations grouped into piRNA, snoRNA, tRNA, other sncRNA, mRNA, and lncRNA classes, multi-mapping status, and a unique sequence identifier based on the MINTplate format (Pliatsika *et al.* 2016). For tRNA-derived fragments, the official fragment name reported by tDRnamer is also provided (Holmes *et al.* 2023). An example of the output quantification file is provided in Table S4, available as supplementary data at *Bioinformatics Advances* online.

### 2.3 Reference databases

Accurate annotation of small RNA-seq data depends on the quality and consistency of the reference databases used. In practice, partially overlapping sncRNA reference resources exist that differ in format, coverage, and annotation standards. Many databases provide only RNA sequences without genomic coordinates, while others report genomic loci using inconsistent identifiers or naming conventions.

To address these limitations, PerSeqPIPE integrates multiple well-established databases for each RNA class (see Table S5, available as supplementary data at *Bioinformatics Advances* online), collects and cleans their sequences, assigns all compatible genomic loci, and compiles the annotations into a unified genome-coordinated GTF. Figure S1, available as supplementary data at *Bioinformatics Advances* online, illustrates this process for piRNA annotation, including integration of multiple reference resources, sequence deduplication, assignment of genomic loci, and retention of compatible annotations. All reference-generation scripts are documented in the PerSeqPIPE GitHub repository, with raw source data available through Zenodo to support reproducibility, transparent updates, and reuse.

### 2.4 Visualization of PerSeqPIPE results

To facilitate interpretation of PerSeqPIPE results, we developed PerSeqPIPE VIZ, a web application for interactive exploration of pipeline outputs. It includes four modules that cover visualization of read statistics, sequence-level sncRNA counts, differential expression results, and genomic coordinates and alignments for selected sequences (e.g. visualizations see Figs. S3–S6, available as supplementary data at *Bioinformatics Advances* online).

### 2.5 Application

To illustrate the additional biological insight provided by sequence-resolved analysis, we reanalyzed the dataset from Gregorova *et al.* 2025 (GSE262424), which profiled miRNAs in bone marrow plasma extracellular vesicles from patients with multiple myeloma, extramedullary disease, and plasma cell leukemia. The original study focused on gene-level miRNA quantification and reported 1,128 miRNAs, including 42 significantly deregulated among the analyzed subgroups (*P* < .05).

PerSeqPIPE further resolved which specific isomiRs contributed to the expression of canonical miRNAs reported in the original study. Specifically, hsa-miR-143-3p, validated by RT-qPCR, displayed three distinct isomiRs (adjusted *P*-value < .05), with one predominant isoform (hsa-miR-143-3p; iso_3p: c) accounting for most of the signal (see Fig. S2, available as supplementary data at *Bioinformatics Advances* online). Two other validated miRNAs, hsa-miR-584-5p and hsa-miR-140-3p, also exhibited significant differential expression of specific isomiRs (adjusted *P*-value < .05) rather than their canonical reference sequences. This isoform-resolved quantification enables a more comprehensive characterization of miRNA regulation.

In addition, PerSeqPIPE identified 3,672 distinct sequences (expression ≥20 reads in at least six samples) corresponding to diverse sncRNAs, fragments of mRNA or lncRNA transcripts, and other uncharacterized RNA species. Among these, 23 sequences were significantly differentially expressed between the analyzed conditions (adjusted *P*-value < .05). Only seven were uniquely mapped, whereas 16 were multi-mapped and multi-assigned—reads that would typically be discarded or fractionally counted in conventional gene-centric workflows. Except for three sequences lacking any known annotation, all others could be associated with multiple annotated RNAs and RNA classes. By retaining all compatible genomic and transcriptomic annotations, PerSeqPIPE provides deeper biological insight into ambiguous sncRNA signals and highlights candidate sequences for further investigation and experimental validation with methods such as RT-qPCR.

### 2.6 Comparison with existing small RNA-seq pipelines

To place PerSeqPIPE in the context of existing small RNA-seq analysis tools, we compared its main characteristics with representative pipelines (Table S6, available as supplementary data at *Bioinformatics Advances* online), including supported RNA classes, quantification strategy, handling of multi-assigned reads, reproducibility, execution mode, and workflow-level parallelization. PerSeqPIPE is distinguished by combining broad sncRNA profiling with sequence-centric quantification, retention of all compatible annotations, and a containerized Nextflow implementation enabling reproducible and parallel execution.

## 3 Conclusion

PerSeqPIPE provides a reproducible, sequence-centric framework for comprehensive small RNA-seq analysis that extends beyond miRNAs and isomiRs to include diverse sncRNA classes. By quantifying each unique read and retaining all compatible annotations, PerSeqPIPE preserves information from multi-mapping reads that is commonly lost in gene-centric workflows. Implemented in Nextflow with containerized dependencies, PerSeqPIPE supports portable execution without extensive software installation. A key component of PerSeqPIPE is a precompiled, genome-coordinated GTF reference that integrates multiple sncRNA databases, providing a ready-to-use reference while still supporting user-provided custom annotations. PerSeqPIPE VIZ further supports interactive exploration, filtering, and visualization of results. Together, they provide an open, scalable, and transparent solution for reproducible small RNA-seq analysis.

## Supplementary Material

vbag294_Supplementary_Data

## Data Availability

Results from the GSE262424 analysis are available at https://doi.org/10.5281/zenodo.22768644.
