# Supplementary material for: PerSeqPIPE: Nextflow workflow for per-sequence analysis of small RNA-sequencing data
Source: Bioinform Adv. 2026 Oct 5;6(1):vbag294. doi: 10.1093/bioadv/vbag294 (PMC13676317; doi:10.1093/bioadv/vbag294)
Supplement: Supplementary file 1 [file vbag294_supplementary_data.zip › Supplementary Information.pdf]

Supplementary Information

PerSeqPIPE: Nextflow workflow for per-sequence analysis of small RNA-sequencing data

Karolína Trachtová[1,2], Dagmar Al Tukmachi[1], Vojtěch Bystrý[1], Ondřej Slabý[1,3,4]

[1] Central European Institute of Technology, Masaryk University, Kamenice 5, 625 00 Brno, Czech Republic

[2] National Centre for Biomolecular Research, Faculty of Science, Masaryk University, Kamenice 5, 625 00 Brno, Czech Republic

[3] Department of Biology, Faculty of Medicine, Masaryk University, Kamenice 5, 625 00 Brno, Czech Republic

[4] Center for Precision Medicine, University Hospital Brno, Jihlavská 20, 602 00 Brno, Czech Republic.

Content

Supplementary Tables

- **Supplementary Table S3**: Computational performance and scalability of PerSeqPIPE
- **Supplementary Table S6**: Comparison of representative small RNA-sequencing analysis pipelines

Supplementary Figures

- **Supplementary Figure S1**: Overview of piRNA annotation and quantification process in PerSeqPIPE
- **Supplementary Figure S2**: Example of isomiR annotation, quantification and differential expression analysis in PerSeqPIPE
- **Supplementary Figure S3**: Reads Statistics Viewer, subsection Reads Statistics Visualization
- **Supplementary Figure S4**: Example of section sncRNA Counts Viewer
- **Supplementary Figure S5**: DE Analysis Viewer, subsection Heatmap sequence selection
- **Supplementary Figure S6**: DE Analysis Viewer, subsection Heatmap appearance

Supplementary Table S3. *Computational performance and scalability of PerSeqPIPE.*

Samples	Runtime	Time per sample	Peak CPU usage	Peak RAM usage (GB)	Storage (GB)		
					outdir	workdir	reference
1	3.7 min	3.7 min	6.6 cores	12	0.2	0.4	15
5	4.4 min	0.9 min	21.0 cores	45	1.1	1.9	15
10	6.8 min	0.7 min	23.7 cores	44	2.0	3.6	15
20	10.9 min	0.5 min	25.7 cores	45	3.9	7.0	15

Runtime and computational resource usage were evaluated using batches of 1, 5, 10, and 20 samples, each subsampled to 10 million reads, on a system equipped with 36 CPU cores and 64 GB RAM. PerSeqPIPE was executed using default pipeline parameters. Increasing the batch size from 1 to 20 samples resulted in an approximately threefold increase in total runtime, demonstrating efficient utilization of Nextflow-based parallel processing across samples. Peak CPU and RAM values represent the maximum concurrent resource usage observed during pipeline execution rather than the total resources available on the system. Because Nextflow schedules tasks according to workflow dependencies and predefined per-process resource requests, additional CPU cores or memory may remain unused when no further tasks can be executed concurrently. Reported runtimes do not include the one-time download of reference resources required when the pipeline is executed for the first time.

Supplementary Table S6. *Comparison of representative small RNA-sequencing analysis pipelines.*

	RNA classes			Quantification unit	Multi-assigned reads strategy	Reproducibility	Execution / deployment			Workflow-level parallelization
	miRNA/ isomiRs	other sncRNA	mRNA/lncRNA fragments				Local	Web-server	Containerized	
**PerSeqPIPE (2.0.0)**	✓	✓	✓	**unique sequence**	**count once per sequence; all annotations retained**	**high (versioned workflow + dependencies)**	✓	✘	✓	✓
**nf-core smrnaseq (2.4.1)**	✓	✘	✘	RNA feature	multiple assignment	**high (versioned workflow + dependencies)**	✓	✘	✓	✓
**sRNAtools**	✓	✓	✓	RNA feature	single assignment; predefined RNA-class priority	medium (no versioned workflow release)	✓	✓	✓	✘
**sRNAtoolbox**	✓	✓	✓	RNA feature	multiple / fractional / single assignment	medium (no versioned workflow release)	✓	✓	✓	✘
**COMPSRA (1.0.3)**	✓	✓	✘	RNA feature	multiple assignment	low (versioned workflow; external dependencies required)	✓	✘	✘	✘
**Oasis 2**	✓	✓	✘	RNA feature	not specified	low (no versioned workflow release)	✘	✓	✘	✘
**docker4seq (1.0.0)**	✓	✓	✘	RNA feature	single assignment	**high (versioned workflow + dependencies)**	✓	✘	✓	✘

RNA classes indicate the major RNA categories directly quantified by each pipeline. **Quantification unit** defines the primary entity for which abundance is reported, distinguishing sequence-centric from traditional gene-centric approaches where reads are assigned to RNA features. **Multi-assigned reads strategy** summarizes the handling of reads compatible with multiple annotations: *multiple assignment* allows a read to contribute to more than one compatible feature; *single assignment* assigns it to one feature according to a tool-specific rule; *fractional assignment* distributes its count among compatible features; and

RNA-class priority resolves assignments between RNA classes according to a predefined hierarchy. **Reproducibility** reflects the availability of versioned software/workflows and their computational dependencies; *high* indicates that both workflow/software and dependencies are reproducibly defined, *medium* indicates that a containerized computational environment is available but no versioned workflow release is provided, and *low* indicates incomplete workflow and/or environment versioning. **Execution/deployment** indicates whether analysis can be performed locally, through a hosted web server, and/or using a containerized environment. **Workflow-level parallelization** indicates automatic orchestration of independent workflow tasks and/or samples by a workflow framework, distinct from multithreading within an individual program. Pipeline characteristics were assessed from the publications listed in References section below and official software documentation for the versions indicated where applicable

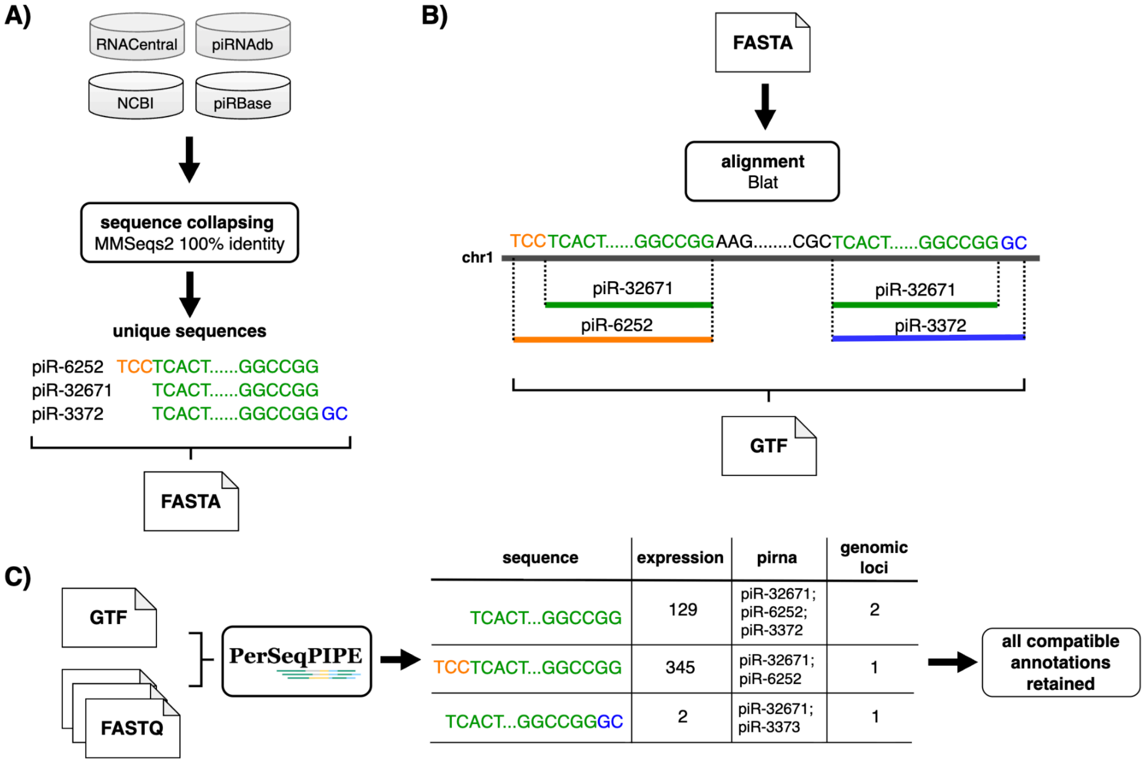

Supplementary Figure S1. *Overview of piRNA annotation and quantification process in PerSeqPIPE.* **(A) Compilation of the piRNA reference.** piRNA sequences are collected from multiple resources (RNAcentral, NCBI, piRNAdb and piRBase) and clustered with MMseqs2 at 100% sequence identity to remove exact duplicates while retaining all distinct sequence variants. **(B) Generation of genomic annotation.** The unique piRNA sequences are aligned to the reference genome using BLAT and converted into a GTF annotation. The schematic example illustrates a key challenge of piRNA analysis: closely related piRNAs may share the same core sequence while differing only by terminal extensions and may map to multiple genomic loci. **(C) Sequence-centric quantification and annotation retention.** Due to the short length and sequence similarity of piRNAs, reads may be compatible with multiple piRNA annotations and genomic loci, making their exact genomic origin difficult to determine from the sequencing read alone. PerSeqPIPE therefore counts each unique read sequence once and retains all compatible piRNA annotations together with the number of genomic loci to which the sequence maps. Rather than assigning ambiguous reads to an arbitrary single piRNA or locus, the complete annotation context is preserved for transparent downstream interpretation and manual inspection.

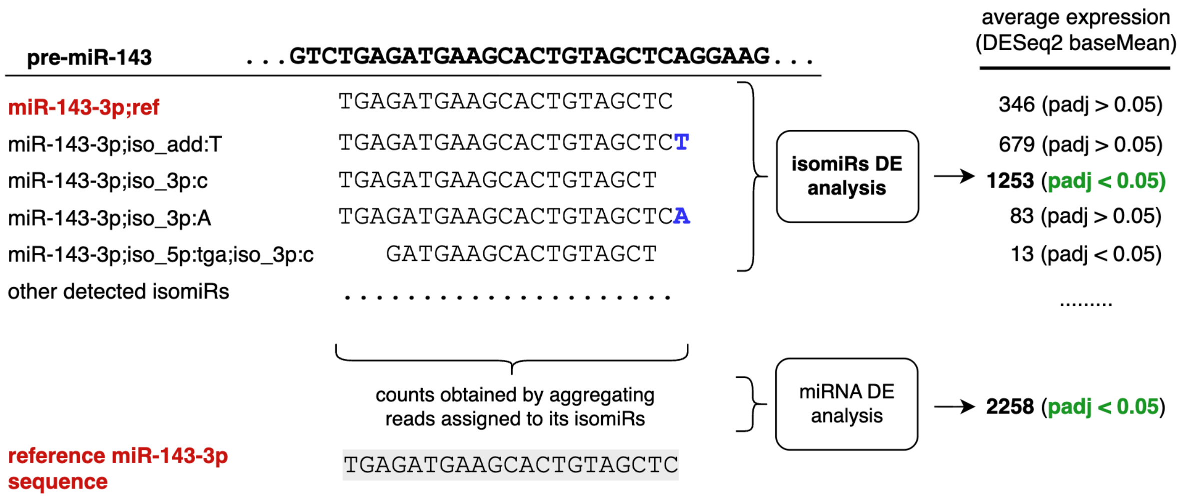

Supplementary Figure S2. *Example of isomiR annotation, quantification and differential expression analysis in PerSeqPIPE.* The schematic shows the pre-miR-143 precursor, reference mature miR-143-3p sequence, and representative detected isomiRs with 3′ additions, 3′ trimming, and combined 5′/3′ end variation. PerSeqPIPE quantifies both individual isomiRs and aggregated mature miRNA expression, enabling differential expression analysis at both levels. In this example, the most abundant isoform is not the reference sequence but the 3′-trimmed isomiR miR-143-3p;iso_3p:c (baseMean = 1253), which is also significantly differentially expressed. In contrast, several other isomiRs contributing to the aggregated miR-143-3p count, including the reference sequence, are not significantly differentially expressed individually. Thus, in some cases aggregated miRNA expression can mask substantial heterogeneity in the abundance and differential-expression patterns of its constituent isomiRs, while isomiR-resolved analysis provides additional sequence-level information.

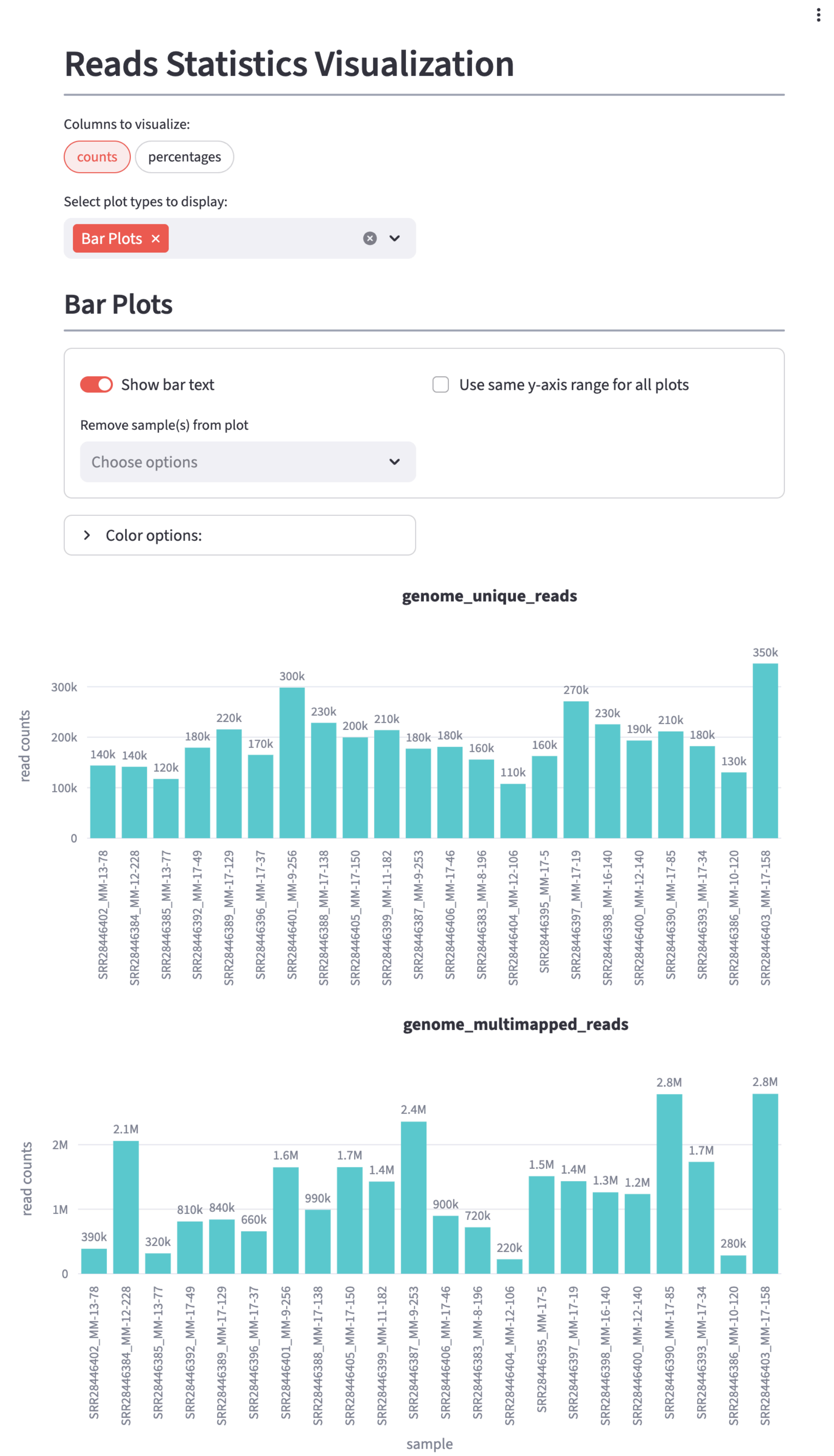

Supplementary Figure S3: *Reads Statistics Viewer, subsection Reads Statistics Visualization.*

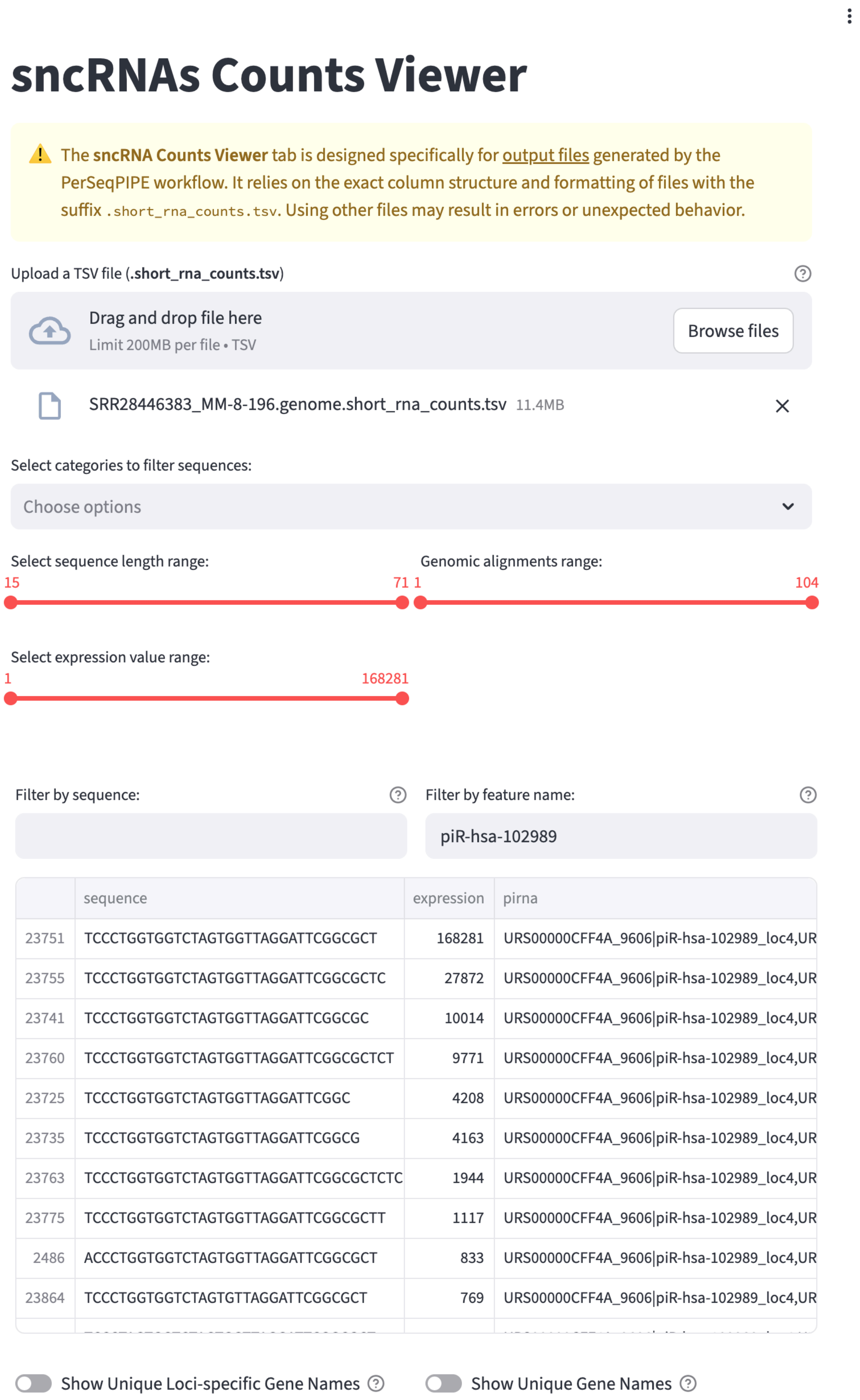

Supplementary Figure S4: *Example of section sncRNA Counts Viewer.*

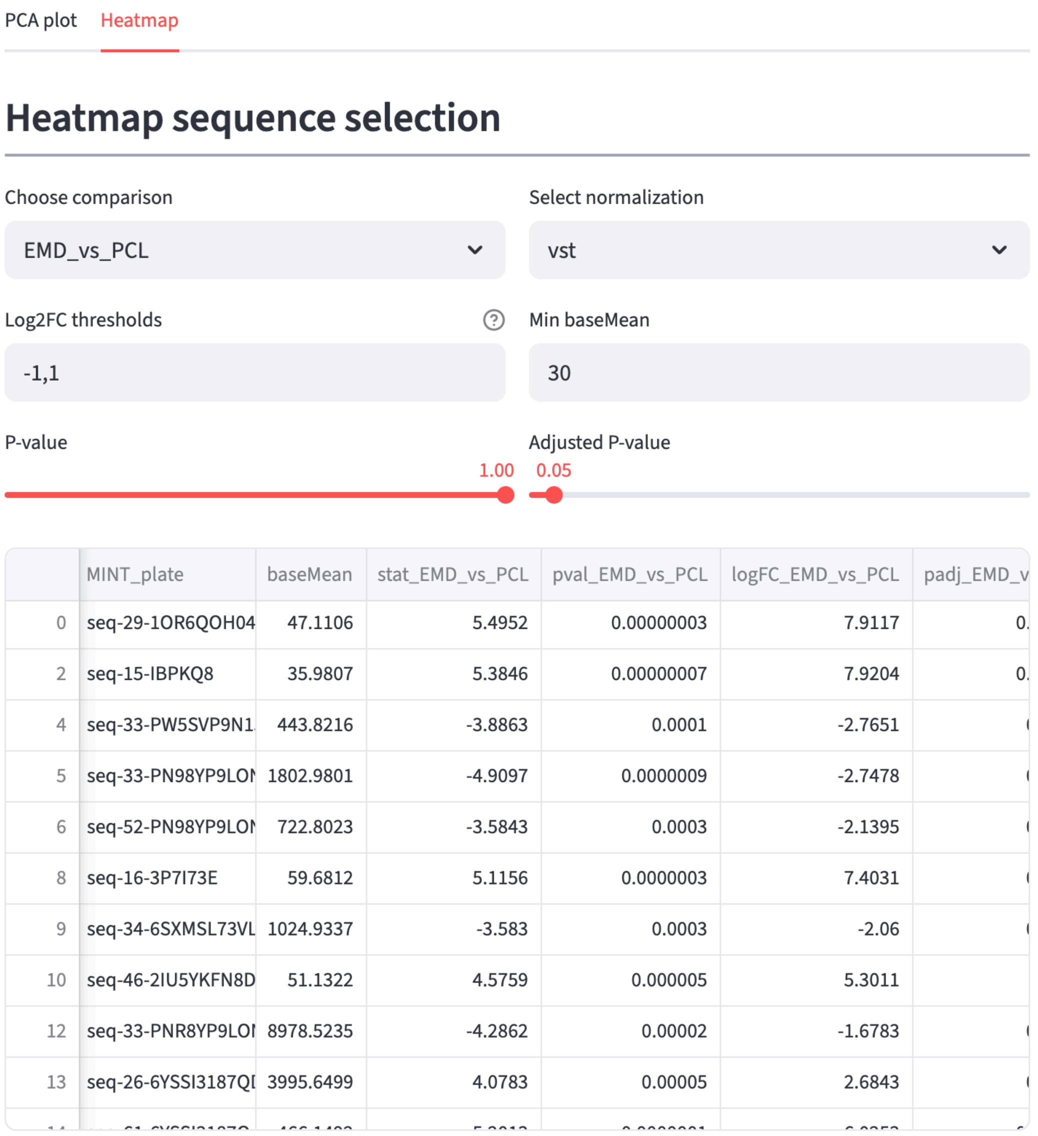

Supplementary Figure S5: *DE Analysis Viewer, subsection Heatmap sequence selection.*

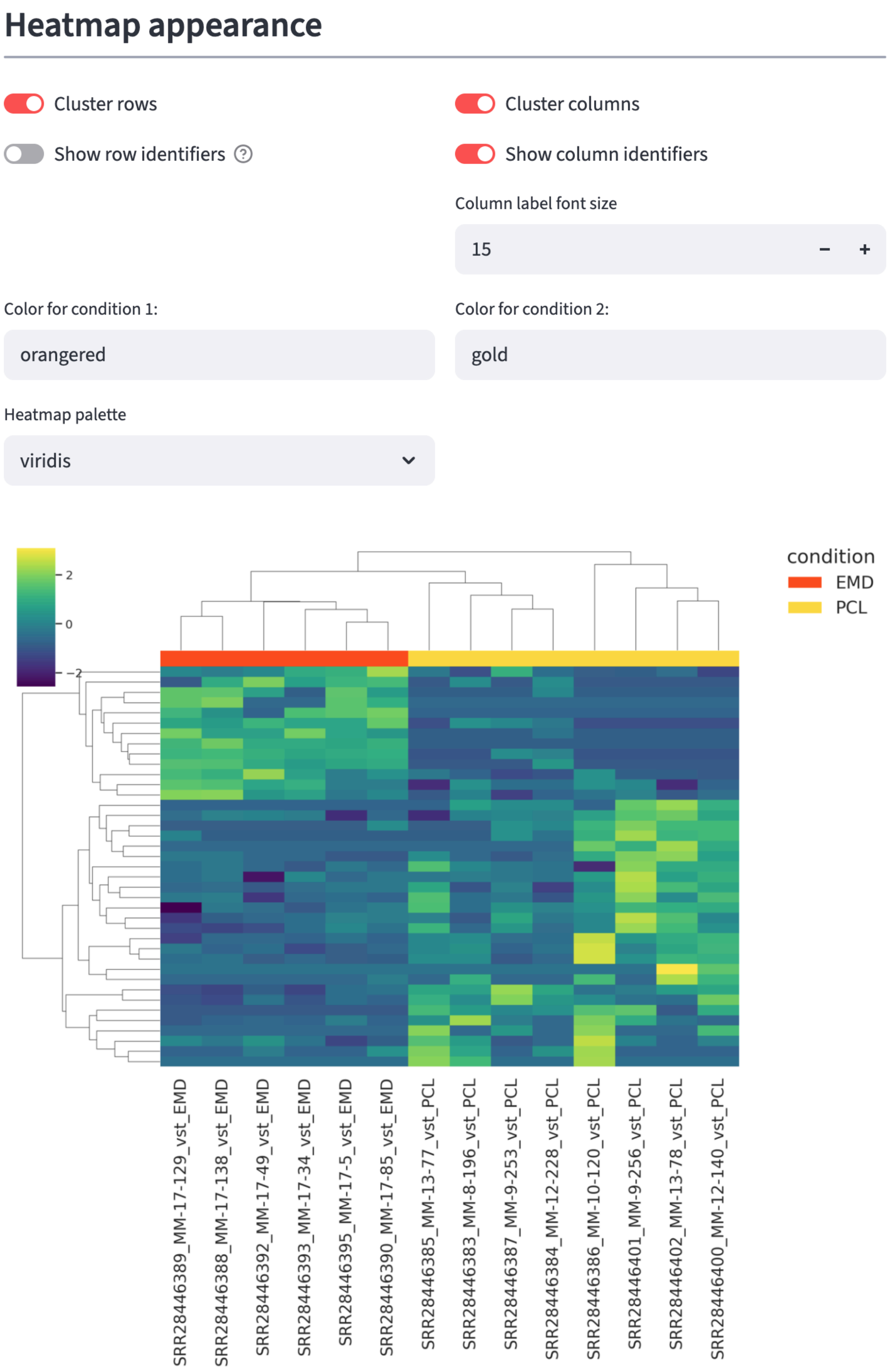

Supplementary Figure S6: *DE Analysis Viewer, subsection Heatmap appearance.*

References

nf-core/smrnaseq. A small-RNA sequencing analysis pipeline. Zenodo. doi:10.5281/zenodo.3456879.

Liu Q, Ding C, Lang X, Guo G, Chen J, Su X. Small noncoding RNA discovery and profiling with sRNAtools based on high-throughput sequencing. Brief Bioinform. 2021;22(1):463-473. doi:10.1093/bib/bbz151

Rueda A, Barturen G, Lebrón R, et al. sRNAtoolbox: an integrated collection of small RNA research tools. Nucleic Acids Res. 2015;43(W1):W467-W473. doi:10.1093/nar/gkv555

Li J, Kho AT, Chase RP, et al. COMPSRA: a COMprehensive Platform for Small RNA-Seq data Analysis. Sci Rep. 2020;10(1):4552. Published 2020 Mar 12. doi:10.1038/s41598-020-61495-0

Rahman RU, Gautam A, Bethune J, et al. Oasis 2: improved online analysis of small RNA-seq data. BMC Bioinformatics. 2018;19(1):54. Published 2018 Feb 14. doi:10.1186/s12859-018-2047-z

Kulkarni N, Alessandrì L, Panero R, et al. Reproducible bioinformatics project: a community for reproducible bioinformatics analysis pipelines. BMC Bioinformatics. 2018;19(Suppl 10):349. Published 2018 Oct 15. doi:10.1186/s12859-018-2296-x
